# Aging Affects the Transcriptional Regulation of Human Skeletal Muscle Disuse Atrophy

**DOI:** 10.1371/journal.pone.0051238

**Published:** 2012-12-19

**Authors:** Charlotte Suetta, Ulrik Frandsen, Line Jensen, Mette Munk Jensen, Jakob G. Jespersen, Lars G. Hvid, Monika Bayer, Stine J. Petersson, Henrik D. Schrøder, Jesper L. Andersen, Katja M. Heinemeier, Per Aagaard, Peter Schjerling, Michael Kjaer

**Affiliations:** 1 Institute of Sports Medicine and Center for Healthy Aging, Faculty of Health Sciences, University of Copenhagen, Bispebjerg Hospital, Copenhagen, Denmark; 2 Institute of Exercise Physiology and Clinical Biomechanics, University of Southern Denmark, Odense, Denmark; 3 Department of Clinical Pathology, Odense University Hospital, Odense, Denmark; 4 Department of Clinical Physiology and Nuclear Medicine, Glostrup Hospital, University of Copenhagen, Copenhagen, Denmark; University of Las Palmas de Gran Canaria, Spain

## Abstract

Important insights concerning the molecular basis of skeletal muscle disuse-atrophy and aging related muscle loss have been obtained in cell culture and animal models, but these regulatory signaling pathways have not previously been studied in aging human muscle. In the present study, muscle atrophy was induced by immobilization in healthy old and young individuals to study the time-course and transcriptional factors underlying human skeletal muscle atrophy. The results reveal that irrespectively of age, mRNA expression levels of MuRF-1 and Atrogin-1 increased in the very initial phase (2–4 days) of human disuse-muscle atrophy along with a marked reduction in PGC-1α and PGC-1β (1–4 days) and a ∼10% decrease in myofiber size (4 days). Further, an age-specific decrease in Akt and S6 phosphorylation was observed in young muscle within the first days (1–4 days) of immobilization. In contrast, Akt phosphorylation was unchanged in old muscle after 2 days and increased after 4 days of immobilization. Further, an age-specific down-regulation of MuRF-1 and Atrogin-1 expression levels was observed following 2 weeks of immobilization, along with a slowing atrophy response in aged skeletal muscle. Neither the immediate loss of muscle mass, nor the subsequent age-differentiated signaling responses could be explained by changes in inflammatory mediators, apoptosis markers or autophagy indicators. Collectively, these findings indicate that the time-course and regulation of human skeletal muscle atrophy is age dependent, leading to an attenuated loss in aging skeletal muscle when exposed to longer periods of immobility-induced disuse.

## Introduction

Skeletal muscle wasting is a common debilitating condition associated with human immobilization and aging resulting in a reduced muscle function [1], [2]. In animal models, loss of muscle mass with immobilization or unloading has been suggested primarily to occur through an accelerated degradation of myofibrillar proteins via the ubiquitin-proteasome pathway [3]–[6], although rapid decreases in protein synthesis also has been shown [7], [8]. Somewhat in contrast, studies in young human individuals have suggested that a decline in protein synthesis rather than accelerated protein breakdown is responsible for the muscle loss observed with disuse [9]–[11]. With aging, muscle loss is suggested to be associated with increased inflammation [12], decreased anabolic signaling [13], increased apoptosis [14], [15], impaired myogenic responsiveness [16], [17] as well as decreased mitochondrial function [18]. Moreover, aging has been found to affect signaling pathways that regulate myogenic growth factors and myofibrillar protein turnover in skeletal muscle of rodents [19], [20]. However, the differential involvement and time course of such signaling pathways remains undescribed in elderly humans exposed to immobilization.

We therefore set to investigate the modulation in cellular signaling pathways involved in the initiation and temporal development of human disuse muscle atrophy, and specifically examine if aging affects the molecular regulation of human disuse related muscle loss. Recent data from our group indicate that, although immobility induces muscle atrophy in both young and old individuals, the loss in muscle mass was more pronounced in young [21], as also demonstrated in rodent models [22]. An age-specific regulation of the signaling pathways orchestrating the initiation and time-course of human disuse muscle atrophy was therefore hypothesized and a range of genes from signaling pathways previously demonstrated to play a central role in the regulation of skeletal muscle atrophy and hypertrophy in a variety of animal models was profiled [4], [6], [23]–[32].

From the ubiquitin-dependent proteolytic system expression levels of Muscle-specific muscle Ring Finger 1 (MuRF-1) and Atrogin-1 was assessed as they have been demonstrated to play a key role in the induction of muscle atrophy in multiple animal disuse models [4], [5], [26], although data from human in vivo studies have been less consistent [9], [33]–[36]. As aging and muscle loss is associated with a decrease in the activation and sensitivity of the IGF-1/Akt signaling pathway [37] gene expression profiles of Insulin-like Growth Factor 1 Ea (IGF-1Ea) and Mechano growth factor (MGF: IGF-1Ec) were assessed, along with protein levels of total and phosphorylated Akt as well as total and phosphorylated ribosomal protein S6. Furthermore, since autophagy in parallel with proteolysis, has been demonstrated to be an important stimulator of muscle atrophy in animal models [25], [32], [38], isoforms of the FoxO family (FoxO3 and possibly FoxO1) along with markers of autophagy (GABARAPL, ATG4 and microtubule-associated protein 1 light chain 3 beta, MAP1LC3B) [39], [40] were examined. Further downstream, a range of genes of importance for oxidative phosphorylation and glycolysis are known to be coordinately suppressed in a variety of models for muscle wasting in rodents [6], [27] and recently also in young human individuals following short term immobilization [35]. Over expression of two of the master genes of mitochondrial biogenesis, peroxisome proliferator-activated receptor gamma co-activator 1 alpha (PGC-1α) and the close homolog PGC-1β, has been shown to prevent muscle atrophy by inhibiting muscle proteolysis [41], and the expression levels of PGC-1α and PGC-1β were therefore assessed to investigate the potential age-specificity of this signaling pathway in human disuse muscle atrophy.

Although, the importance of apoptosis in human skeletal muscle atrophy has been regarded as controversial, we investigated the importance of this pathway by assessing the expression levels of the Bcl-2–associated X protein (Bax), Bcl-2-like protein 1 (BCL2L1) and tumor protein 53 (p53), as apoptosis seems to play an important role in the development of muscle atrophy in aged animal models [14], [42]–[46]. Furthermore, the mRNA expression level of Nuclear Factor of kappa light polypeptide gene enhancer in B-cells 1 (NF-κB) along with the upstream pro-inflammatory cytokine Tumor Necrosis Factor α (TNF-α) were profiled to study the effect of immobility-induced disuse on the induction of the NF-κB pathway [24]. In addition, expression levels of the pro-inflammatory cytokine IL-6 was profiled as an elevated expression of this cytokine along with an increased expression level of TNF-α has been linked to various diseases as well as aging [47].

Collectively, these transcriptional data were combined with measures of contractile capacity, morphology of the immobilized muscle and protein quantification in order to gain a more thorough understanding of the pathways regulating muscle protein degradation with disuse in old versus young human adults and further to examine the influence of these molecular regulatory pathways on muscle function and muscle size.

## Materials and Methods

### Study design

**Figure 1:**
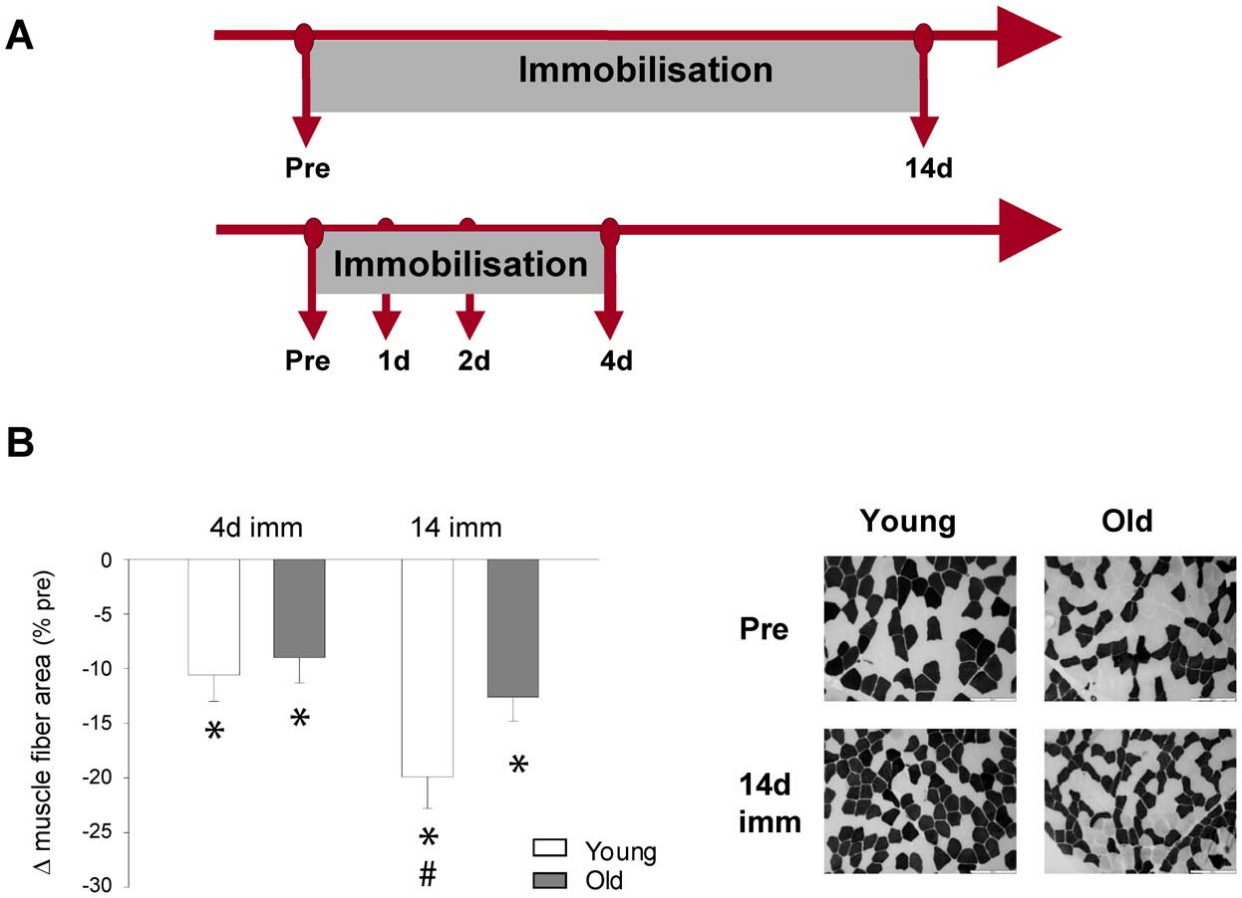
Immobility-induced skeletal muscle atrophy causes an age-specific decline in muscle size. **A.** Scheme of experimental setup, including the time points of muscle biopsy procedure. **B**. Percentage decreases in muscle size (mean muscle fiber area) after 4 days of immobility in young (n = 11) and old (n = 9) as well as after 14 days of immobilization in young (n = 11) and old (n = 12), respectively. * Time effect, p<0.05 compared to pre, # Age effect, p<0.05 young compared to old within time point. Group mean data ± SEM. Mean myofiber area was assessed in the quadriceps femoris muscle, by muscle biopsy sampling. **C.** Muscle histology from resting state (pre) and immobility (14 d) was analyzed by myofibrillar ATPase at pH 10.3 preincubations demonstrating type I (white) and type II muscle fibers (black) [52].

In the present manuscript results from two human intervention studies are reported. At first, myofiber atrophy was induced for a period of 2 weeks to investigate the signaling pathways regulating disuse skeletal muscle atrophy in young and aged individuals, respectively (Figure 1). Muscle biopsies of the vastus lateralis muscle were collected prior to the intervention and immediately after cast removal. In order to study the time-course and identify signaling pathways involved in the initiation of disuse muscle atrophy, two additional groups of young and aged individuals were recruited and immobility was induced for 4 days. These subjects were biopsied after 24 h (∼1 d), 48 h (∼2 d) and 96 h (∼4 d) of immobility (Figure 1A). Within age groups, subjects recruited for the two immobilization protocols did not differ with respect to age, weight, BMI and activity level (Table S1).

### Human subjects

A total of 43 healthy men volunteered to participate in the two immobilization studies, 20 persons were recruited for the 14 d immobilization protocol (11 young: 24.4, range 21–27 yrs; 9 old: 67.3, range 61–74 yrs) while 23 persons were recruited for the 4 d immobilization protocol (11 young: 24.3, range 21–30 yrs; 12 old: 66.8, range 60–72 yrs) (Table S1). Prior to inclusion a trained physician screened all subjects to exclude individuals with cardiovascular disease, diabetes, neural or musculoskeletal disease, inflammatory or pulmonary disorders or any known predisposition to deep venous thrombosis. The local Ethics Committee of Copenhagen and Frederiksberg municipality approved the study (KF01-322606) and all experimental procedures were performed in accordance with the Declaration of Helsinki. Written informed consent was obtained from all participants before inclusion in the study.

### Immobilization protocols

#### 14 d immobilization

This protocol has been described in detail before [21]along with results on muscle contractile properties and morphology [21], [48] but in brief immobilization was accomplished by 2 weeks of randomized unilateral whole-leg casting using a lightweight fiber cast applied from just above the malleoli to just below the groin. The cast was positioned in 30° of knee joint flexion to circumvent walking ability of the casted limb and the subjects were carefully instructed to perform all ambulatory activities on crutches and abstain from ground contact as well as performing isometric contractions of quadriceps of the immobilized leg. Muscle biopsies were obtained at rest 7 days before the intervention (pre) and immediately following removal of the cast 14 days post immobilization (14 d) (Figure 1A).

#### 4 d immobilization

In order to obtain muscle biopsies throughout the immobilization period these subjects had a randomly assigned leg immobilized using a knee brace (DonJoy, Orthopedics, Sunny Vista, CA, US) fixated at a knee angle of 30°. As with the 14 d protocol subjects were provided with crutches during the immobilization period. Muscle biopsies were obtained 1 wk prior to the immobilization (pre), 24 h post immobilization (1 d), 48 h post immobilization (2 d) and 96 h post immobilization (4 d). At all time points muscle biopsies were obtained at rest, and the 1 d and 2 d time point biopsies were obtained without removal of the brace, whereas the 4 d time point biopsies were taken immediately after brace removal.

### Assessment of contractile muscle strength

Maximal muscle contraction strength was measured for the quadriceps muscle in a custom made setup where the subjects were seated in an upright position with back support and the hip and knee joint flexed at 90° [49]. A steel cuff was strapped around the lower leg, approximately 2 cm above the medial malleoli and was connected via a rigid steel bar to a strain gauge load cell (Bofors KRG-4, Bofors, Sweden), which was connected to an instrumentation-amplifier (Gould 5900, Gould Inc. Valley View, OH USA).

### Muscle biopsy sampling and analyses

Subjects were prohibited to exercise at least 2 days before the first biopsy and all subjects were biopsied at the same time of the day (+/−1 h) at each testing session. Biopsies were obtained from the middle portion of m. vastus lateralis utilizing the percutaneous needle biopsy technique of Bergström [50]. All biopsies were obtained by the same investigator, and careful efforts were made to extract tissue from the same depth and within ∼2–3 cm distance between each biopsy, which has been shown to be sufficient to avoid the influence of muscle damage potentially induced by repeated biopsies sampling [51]. After dissecting the muscle samples of all visible blood, adipose and connective tissue, the muscle samples were divided into two separate pieces, one oriented in embedding medium (Tissue Tec) frozen in isopentane cooled with liquid nitrogen and stored at −80°C and one piece directly frozen in liquid nitrogen and stored at −80°C until further analyses. Subsequently, serial transverse sections (10 µm) were cut in a cryotome at −20°C and stained for myofibrillar ATPase at pH 9.4 after both alkaline (pH 10.3) and acid (pH 4.3) preincubations [52]. All samples of each individual person were stained in the same batch to avoid interassay variation. Based on the ATPase staining pattern muscle fibers were characterized as type I and type II and an average of 277+/−49 fibers were analyzed in each biopsy. For the determination of muscle fiber size only horizontally fibers were used, with a minimum of 100 fibers included for the analysis. A videoscope consisting of a microscope (Olympus BX 50) and color video camera (Sanyo high resolution CCD) in combination with Tema Image-analyses System (Scanbeam Denmark) were used to calculate the mean muscle fiber area.

### RNA purification

Total RNA was isolated from ∼20 mg of frozen muscle biopsy by phenol extraction (TriReagent; Molecular Research Center, OH, USA) as previously described [53]. Intact RNA was confirmed by denaturing agarose gel electrophoresis.

### Real-time PCR

Total RNA (500 ng) was converted into cDNA in 20 µl using the OmniScript reverse transcriptase (Qiagen, CA, USA) according to the manufacturer's protocol. The mRNA expression of FoxO1, FoxO3, FoxO4, PGC-1α, PGC-1β, IL-6, MGF, IGF-1Ea, GAPDH and RPLP0 were analyzed by quantitative real-time RT-PCR. For each of the mRNA targets, 0.25 µl cDNA was amplified in a 25 µl SYBR Green PCR reaction containing 1× Quantitect SYBR Green Master Mix (Qiagen) and 100 nM of each primer (Table S2A). The amplification was monitored real-time using the MX3000P real-time PCR machine (Stratagene, CA, USA). The threshold cycle (Ct) values were related to a standard curve made with the cloned PCR products and specificity ensured by melting curves analysis and the quantities were normalized to RPLP0. TaqMan based quantitative real-time RT-PCR of MuRF-1, Atrogin-1, NF-κB, Bax, BCL2L1, p53, TNF-α, ATG4B, GABARAPL1, and RPLP0 mRNA (Table S2B) were performed in the ABI Prism 7900HT Sequence Detection System (Applied Biosystems) using ABI TaqMan Low Density Arrays (Applied Biosystems). Each sample was run in triplicates with 4 samples per card. 250 ng cDNA was mixed with 100 µl 2× TaqMan Gene Expression Mastermix and loaded into two ports (∼2.5 ng cDNA per reaction). Raw data was extracted and analyzed using the SDS 2.1 software (Applied Biosystems) and qBasePlus (Biogazelle) was used to quality-check Ct-values, asses triplicates, exclude runs when the difference among triplicates >0.5 Ct and finally to normalize data to RPLP0 using the 2^−ΔΔCt^ method [54]. To test the use of RPLP0 for normalization, another “housekeeping” mRNA, GAPDH, was measured and normalized with RPLP0 (Figure S4). Unfortunately, the GAPDH to RPLP0 ratio appears to decrease over time, indicating that GAPDH levels decrease or RPLP0 levels increase. As, during inactivity, a decrease in the metabolic mRNA GAPDH seems more likely than an increase in the ribosomal mRNA RPLP0 we selected to use RPLP0 for normalization. It should be noted that if GAPDH, and not RPLP0, were in fact constant other constant mRNA would display a similar pattern as GAPDH in figure S4. Fortunately, none of the other mRNA has this pattern, strengthening our choice of RPLP0 for normalization.

### Immunohistochemistry analysis

Following rehydration, fixation and permeabilisation in 4% normal buffered formalin and triton X-100 (0.1%) for 10 min, double staining were performed on sections from each biopsy by simultaneous incubation with primary antibodies for the basement membrane component laminin rabbit anti-laminin (rabbit anti-laminin, cat. no. Z0097, Dakocytomation) mixed with goat anti-MuRF-1 (cat. no. ab4125, Abcam). Primary antibody binding was visualized with the corresponding secondary antibodies Alexa Fluor 488 donkey anti-rabbit (Molecular Probes A21206) and Alexa Fluor 555 donkey anti-goat (Molecular Probes A A21432), respectively. In order to identify the muscle MHC fiber type expressing of the MuRF-1 protein, goat anti-MuRF-1 was mixed with either mouse anti-skeletal Myosin-Slow or mouse anti-skeletal Myosin Fast (Clone NOQ7.54D; product No. M8421 and clone MY32; product no. M4276, www.sigma-aldrich.com). Primary antibody binding was visualized with Alexa Fluor 555 donkey anti-goat (Molecular Probes A21432) and Alexa Fluor 488 donkey anti-mouse (Molecular Probes A21202). Nuclei were visualized by the addition of 4′,6-Diamidino-2-phenylindole in the mounting medium (Molecular Probes ProLong Gold antifade reagent P36931). The relative number of fibers expressing MuRF-1 was determined arbitrary from approximately 200 fibers analyzed from 3 microscope images (200×). Digital image processing was performed using Axio imager M1 and Axio Vision by Zeiss (www.zeiss.com).

### Determination of nuclear apoptosis

Apoptotic cells were determined by Terminal Deoxynucleotidyl Transferase Biotin-dUTP Nickend Labeling (TUNEL) (In Situ Cell Death Detection Kit, Fluorescent, cat. no. 11 684 795 910, www.roche-applied-science.com) as described by the manufacturer. To determine whether TUNEL-positive nuclei were located inside the muscle membrane or in interstitial cells, each section was fixed and permeabilized with 4% normal buffered formalin and triton X-100 (0.1%) for 10 min. TUNEL fluorescent reaction mix was then added and incubated at 37°C for 60 min in the dark. Subsequently, each section was washed and blocked with protein blocker for 10 min (DakoCytomation cat. no. S0809). The muscle plasmalemma was localized by incubation with goat anti neuronal nitric oxide synthase (anti-human nNOS, cat. no. Af2416, www.RnDsystems.com) and subsequently visualized by incubation with Alexa 555 conjugated donkey anti goat antibody (Molecular probes A41432) for 60 min in the dark. Sections were cover slipped and nuclei was visualized by the addition of 4′,6-Diamidino-2-phenylindole in the mounting medium (Molecular Probes, ProLong Gold antifade reagent, cat. no. P36931). All positive nuclei on each section were counted on microscope images obtained at 200× and the number of TUNEL-positive nuclei located inside or outside the plasmalemma membrane was determined by visualized nNOS expression. Nuclei identified inside the muscle membrane were characterized as myofiber nuclei, while nuclei located outside the muscle membrane were denoted as interstitial nuclei. In order to identify the cellular identity of TUNEL-positive cells double stainings was performed with TUNEL and with primary antibodies against endothelial cells (CD-31, code nr. M 0823, DakoCytomation, Denmark), macrophages (CD68 code nr. M 0814, DakoCytomation, Denmark) and muscle satellite cells (Pax7, DSHB, University of IOWA, USA) and subsequently visualized with corresponding Alexa Flour 555 secondary antibodies. The number of positive nuclei was counted per section and expressed per mm^2^, using the digital image processing software axio vision by Zeiss (www.zeiss.com).

### Western blotting analyses

From each muscle biopsy 150 cryosections (10 µm) were homogenized in a micro vial containing 1 silicium carbide crystal, 5 steel beads (2.3 mm) and 250 µl ice-cold homogenization buffer (50 mM Tris-base, 1 mM EDTA, 1 mM EGTA, 10 mM beta-glycerophosphate, 50 mM sodium fluoride, 0.5 mM sodium orthovanadate, 0.1% v/v, Triton-X, 0.1% v/v mercaptoethanol and protease inhibitor (Complete, Roche, Basel, Schwitzerland), pH 7.5) using a FastPrep-24 (MP Biomedicals, Solon, OH, USA) homogenizer. Laemmli buffer was added and protein concentrations were determined with the EZQ Protein Quantitation Kit according to the manufacturer's protocol (Molecular Probes, Eugene, OR, USA). Then, samples were heated at 90°C for 4 min, shortly vortexed and spun in a microcentrifuge and equal amounts (10 µg/4 µl) were separated by SDS-PAGE using a 4–12% Bis-Tris gel (Criterion, Bio-Rad, Hercules, CA, USA) at 200 V for 1 h. Gels were blotted (Trans-blot cell, Bio-Rad, 400 mA, 2 h) to polyvinylidene difluoride membranes (Amersham Hybond LFP, GE Healthcare, Buckinghamshire, UK), which were blocked for 30 min with 20% Odyssey blocking buffer (Li-Cor Biosciences, Lincoln, NE, USA) in phosphate-buffered saline, incubated overnight at 4°C with primary antibody, incubated for 1 h in fluorophore-conjugated with secondary antibody and visualized with the Odyssey Infrared Imaging System (Li-Cor Biosciences). Total and phosphorylated protein pairs were detected simultaneously on the same membrane. Band intensities were quantified using ImageJ (National Institutes of Health, Bethesda, MD, USA). Total and phospho (serine 473) Akt primary antibodies (Cell Signaling Technology, Danvers, MA, USA, no. 2920 and 4060) were diluted 1∶2,000 and actin (Sigma, Saint Louis, MO, USA, no. A2066) primary antibody was diluted 1∶10,000. Due to low tissue availability n equals 3 in each age group for the measurement of total and phospho Akt. Western blot analysis for LC3B, as well as S6 ribosomal protein and phospho-S6 ribosomal protein (Ser235/236) were performed on frozen tissue homogenized in 10 volumes (wt/vol) of ice-cold buffer (300 mM Sucrose, 1 mM EDTA, 10 mM NaN3, 40 mM tris-base and 40 mM histidine at pH 7.8 with protease inhibitors, #05892791001, Roche Inc.) using a 1 ml glass homogenizer with a glass pestle (Kontes Glass Industry, Vineland, NJ). Protein content in the muscle homogenate was measured in triplicates using a standard kit (Pierce BCA protein reagent no. 23225, Pierce Inc.). Samples were heated at 90°C for 4 min, shortly vortexed and spun in a microcentrifuge and equal amounts (20 µg) were separated by SDS-PAGE using a 4–15% Tris/glycine gel (Miniprotean TGX, BioRad, Hercules, CA, USA) at 200 V for 35 min. Gels were blotted (Trans-blot cell, Bio-Rad, 250 mA, 1 h) to polyvinylidene difluoride membranes (Immun-Blot PVDF, 0.2 µm, BioRad, Hercules, Ca, USA), which were blocked for 60 min with 5% Blotting–Grade Blocker (#170-6404, BioRad, USA) in phosphate-buffered saline with 0.05% Tween 20. Membranes were subsequently incubated overnight at 4°C with primary antibody LC3B (1∶1000, #2775, Cell Signaling Technology) as well as S6 ribosomal protein (1∶1000, #2217, Cell Signaling Technology Inc.) and phospho-S6 ribosomal protein (Ser235/236) (1∶2000, #4858, Cell Signaling Technology Inc.). Membranes were subsequently washed and incubated for 1 h with HRP-conjugated secondary antibody (Immun-Star Goat Anti-Rabbit (GAR)-HRP Conjugate, #170-5046, BioRad, Hercules, Ca, USA) and visualized with Immun-star western kit (#170-5070, BioRad, Hercules, Ca, USA). After detecting the phosphorylated protein, the membrane was stripped and the total protein was detected. Band exposure and visualization were quantified using ChemiDoc XRS with Image Lab Software (Bio-Rad Laboratories, Inc.)

### Statistical analyses

A part from total and phosphorylated S6 ribosomal protein, all mRNA and Western blot data were log-transformed prior to statistical analyses and are presented as geometric means ± back-transformed SEM. To test for changes over time (mRNA, TUNEL, Immunohistochemistry and Western blot analyses) one-way Bonferroni corrected repeated-measures ANOVA was performed separately for young and old individuals, respectively, as well as for young and old combined (SPSS). Pair wise multitude comparison procedures were evaluated using Student-Newman-Keuls Method post-hoc testing. Independent-samples t-testing were used to test for differences between groups' with a subsequent Bonferroni correction. As phosphorylated S6 ribosomal protein (but not total S6 ribosomal protein) became non-detectable after immobilization in a high number of especially young subjects, quantification of phosphorylated S6 ribosomal protein/total S6 ribosomal protein was not possible and we therefore decided to perform a Chi-square test (young p<0.001, old p = 0.44). The percentage of the total number of subjects where p-S6 could be detected, at each time-point, are visualized in figure 4D. Non-parametric statistics were used to analyze changes in muscle fiber CSA, since not all of these data were normally distributed. To evaluate the effect of intervention over time a repeated-measures Friedman test was used with post-hoc Wilcoxon testing. Between-group differences were analyzed with Kruskall-Wallis tests and subsequent Mann-Whitney U testing. Data are presented as mean values ± SEM. A p-value of less than 0.05 was considered statistically significant.

## Results

### Maximal contractile muscle strength

Maximal contractile muscle strength declined in both young (13%) and old (14%) after 4 days of unloading, with no difference between age groups (Figure S1A). Following 14 days of unloading maximal isometric muscle strength was reduced by 20% in young and by 16% in old, again with no age-related differences (Figure S1A).

### Muscle fiber cross sectional area

Despite the very limited period of immobilization, our histological analyses revealed significant decreases in mean myofiber area of approximately 10% in both young and old subjects after 4 days of immobility (Figure 1B). However, following 14 days of immobilization the loss in mean myofiber size was greater in young (−19.9%) than old individuals (−12.6%, p<0.01) (Figure 1B). There was no difference between the decline in type 1 (O: −7.1%, Y: −8.1%) and type 2 (O: −10.9%, Y: −12.6%) fibers at the 4 d time-point, however after 14 d the decline in type 2 fibers (O: −17.1%, Y: −26.5%) was significantly larger than in type 1 fibers (O: −8.9%, Y: −14.3%) in both young and old (p<0.05) (Figure S1B).

### Ubiquitin ligases

**Figure 2:**
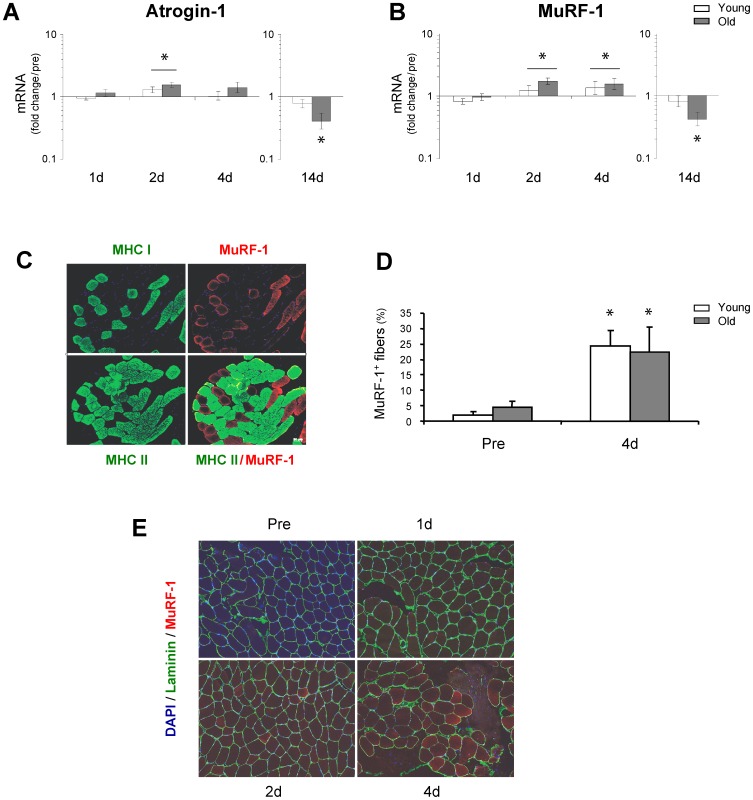
Immobility results in an increase in the transcriptional status and immunodetection of Atrogin-1 and MuRF-1 in the early phase of disuse muscle atrophy. **A & B**. The mRNA level of Atrogin-1 and MuRF-1 was determined using qRT-PCR and significant increases were found in the early phase (2–4 days) of immobility, in both young and old skeletal muscle. At the later time-point (14 d) Atrogin-1 and MuRF-1 expression levels decreased in aged muscle, whereas the expression levels of Atrogin-1 and MuRF-1 mRNA were unchanged from baseline in young muscle. Data are geometric means ± back-transformed SEM. **C**. Immunodetection of DAPI (blue), and MuRF-1 (red) are shown for 10 µm skeletal muscle cryosections. Potential muscle fiber specificity was analyzed by simultaneous incubation of primary antibodies for MuRF-1 mixed with either anti-skeletal Myosin-Fast or anti-skeletal Myosin-Slow (green), demonstrating an almost 100% affinity of MuRF-1 for type 1 muscle fibers. **D**. Total numbers of MuRF-1 positive myofibers were quantified for both young and old muscle and a significant increase was detected in the number of MuRF-1 positive fibers after 4 days of immobility. * Time effect, p<0.05 compared to pre. * Time effect, p<0.05 bar indicates young and old combined compared to pre. Data are means ± SEM. **E**. Immunodetection of DAPI (blue), Laminin (green) and MuRF-1 (red) are shown for 10 µm skeletal muscle cryosections at pre, 1 d, 2 d and 4 d of immobilization.

The present data revealed a rapid up-regulation of MuRF-1 and Atrogin-1 mRNA expression levels after 48 h in both young and old subjects (Figure 2, A–B). These data were further supported by immunohistochemical staining for MuRF-1 (Figure 2C), which demonstrated an acute increase in the number of MuRF-1 positive fibers and nuclei within the first days of immobility in both young and old muscle (Figure 2D). Notably, there was an almost 100% affinity of MuRF-1 for type 1 muscle fibers (Figure 2C), although this was not accompanied by a selective type 1 muscle fiber atrophy at the 4 d time point (Figure S1B).

### IGF-1/Akt signaling

**Figure 3:**
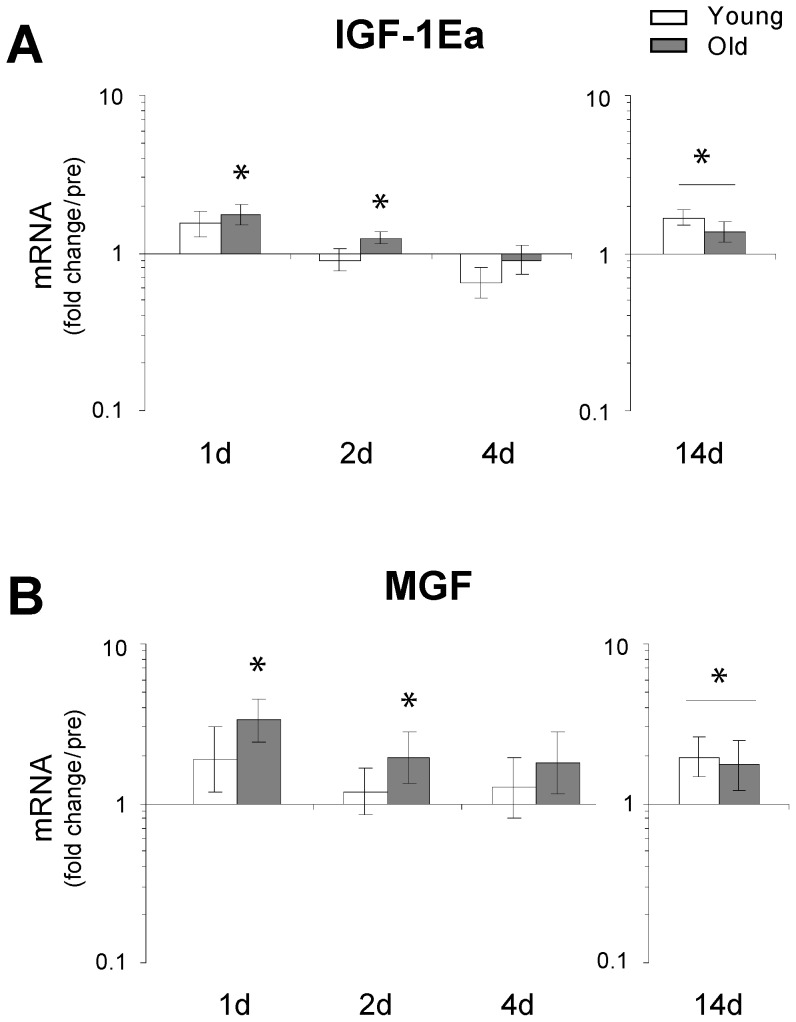
Changes in the transcriptional status of IGF-1Ea and MGF as a result of immobility induced disuse-muscle atrophy. Expression levels of IGF-1Ea and MGF mRNA were determined using qRT-PCR. **A & B**. The data revealed an age-specific (old subjects only) up-regulation of IGF-1Ea and MGF at 1 day and 2 days of immobility while an up-regulation was observed after 14 days of immobility in both age groups. * Time effect, p<0.05 compared to pre. Data are geometric means ± back-transformed SEM.

**Figure 4:**
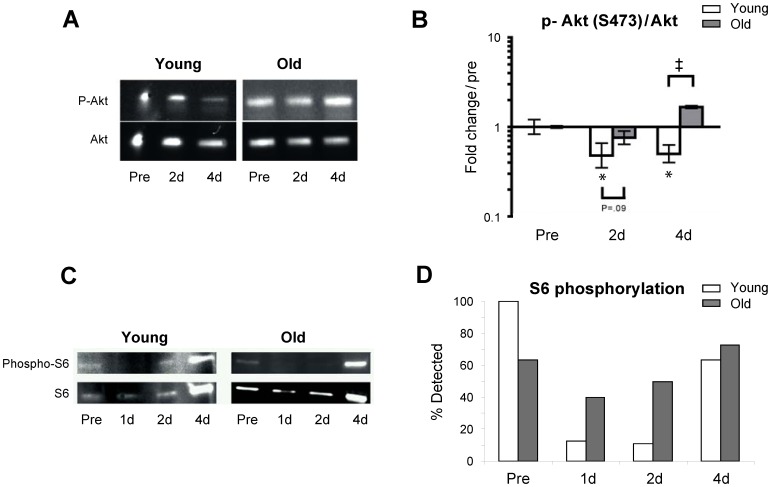
Immobility induced skeletal muscle atrophy results in an age-specific decrease in Akt and ribosomal protein S6 phosphorylation. **A.** Western blotting of whole muscle protein homogenates of phosphorylated Akt and total Akt. **B.** Immobility decreased levels of phosphorylated Akt/total Akt ratio (p-Akt/Akt) at the early (2–4 days) phase of immobility in young but not aged skeletal muscle. * Time effect, p<0.05, compared to pre. # Age effect, p<0.001 young compared to old within time point. Due to lack of muscle tissue n = 6 (3 young and 3 old) in these analyses. **C.** Western blotting of whole muscle protein homogenates of total and phosphorylated S6 ribosomal protein. **D.** The percentage of the total number of subjects at each time point where p-S6 could be detected. Chi-square: Young p<0.001, Old p = 0.44. In a high number of especially young subjects phosphorylated S6 ribosomal protein (but not total S6 ribosomal protein) became non detectable after immobilization which made an exact quantification impossible.

The data revealed an age-specific (old subjects only) up-regulation of IGF-1Ea and MGF at 1 d and 2 d of immobilization, while a general up-regulation was observed in both age groups after 14 days of immobility (Figure 3, A–B). Furthermore, protein analysis by Western blotting revealed selective decreases in phosphorylated Akt/total Akt ratio (2 d and 4 d) in young individuals. In addition, the number of subjects, where phosphorylated S6 ribosomal protein could be detected within the very first days of immobilization was reduced in young compared to old individuals (1 d and 2 d) (Figure 4, A–D). No change was observed in protein levels of Actin in neither young nor old muscle (Figure S3).

### Forkhead box O (FoxO) transcription factors

**Figure 5:**
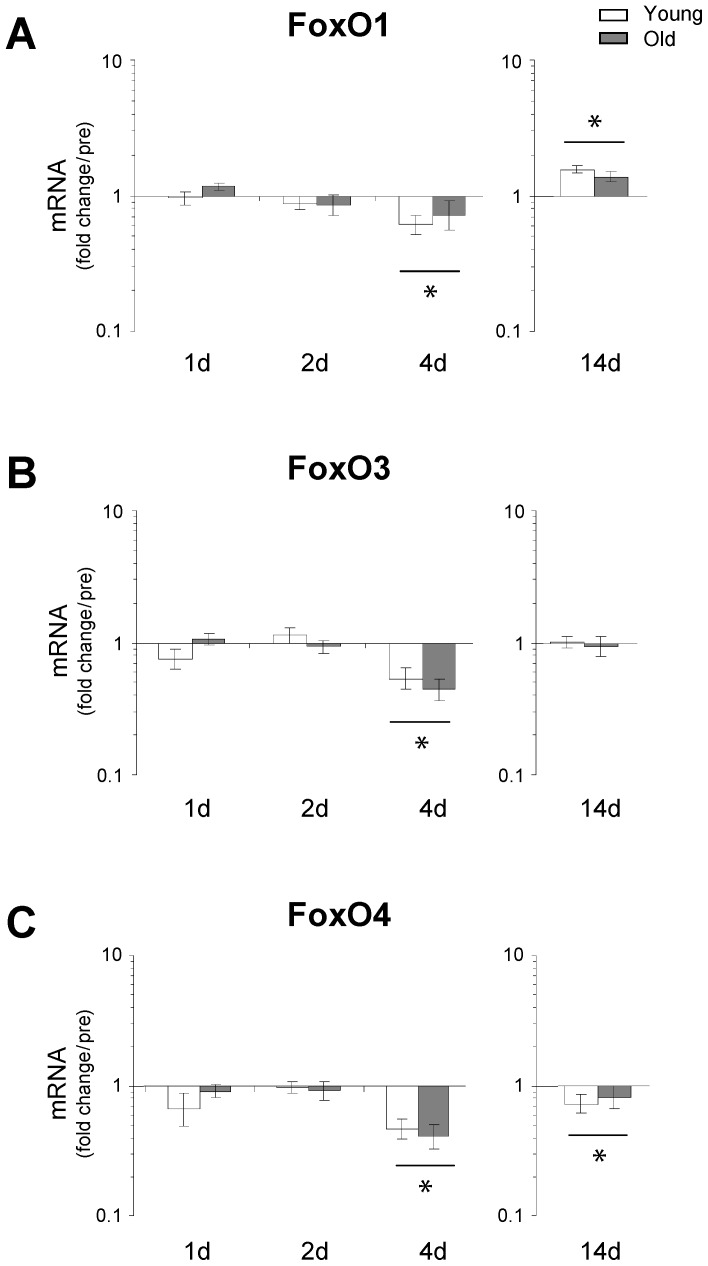
Changes in the transcriptional status of FoxO1, FoxO3 and FoxO4 as a result of immobility-induced muscle disuse. **A–C.** The mRNA level of FoxO1, FoxO3 and FoxO4 was determined using qRT-PCR. No up-regulation in the mRNA expression levels in any of these three genes was observed during the initial phase of immobility, in contrast, a general down-regulation in all three genes was observed in both young and aged muscle at the 4 d time point, potentially reflecting a negative feedback signal from high presence of active FoxO protein in the muscle cell. However, it is difficult to interpret the role of FoxO in the present study since the phosphorylated forms of FoxO were not measured. * Time effect, p<0.05 bar indicates young and old within time point. Data are geometric means ± back-transformed SEM.

There was no up-regulation in the mRNA expression levels of any of the three isoforms of the FoxO family in skeletal muscle (FoxO1, FoxO3 and FoxO4) after 4 days or 14 days of immobilization. In fact, a general down-regulation in all three genes was observed in both young and aged muscle after 4 days of unloading but not at the 14 days time point (Figure 5, A–C). No age related differences were observed at any time point (Figure 5, A–C).

### Autophagy

**Figure 6:**
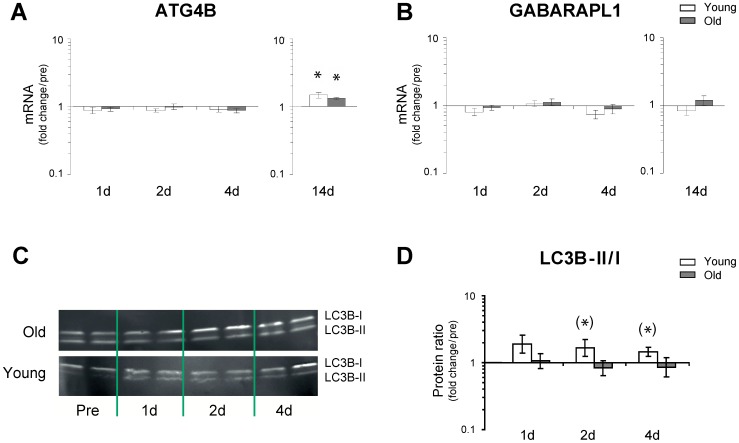
Changes in the transcriptional status of ATG4B and GABARAPL1 and LC3B II/I ratio as a result of immobility induced disuse muscle atrophy. mRNA expression levels of ATG4B and GABARAPL1 were determined using qRT-PCR **A–B**. ATG4B mRNA expression increased in both age groups after 14 days of immobility, otherwise no changes were observed in expression levels of ATG4B and GABARAPL1. * Time effect, p<0.05 compared to pre. Data are geometric means ± back-transformed SEM. **C.** Western blotting of whole muscle protein homogenates of LC3B I and II isoforms; quantified in **D.** Immobility tended to increase levels of LC3B II/I ratio at 1 days (p = 0,093) and at 4 days (p = 0,066) in young but not aged skeletal muscle (compared to pre). N = 18 (9 young and 9 old).

The mRNA expression profiles of cysteine protease ATG4B and GABARAPL1 were examined, and a significant up-regulation of ATG4B was observed after 14 days of immobility in both young and old (Figure 6A), whereas no changes were observed for the expression level of GABARAPL1 (Figure 6B). However, using immunohistochemical targeting of ATG4B no visible autophagosomes could be observed in biopsies obtained from young and old individuals (data not shown). However, protein analysis of microtubule-associated protein 1 Light Chain 3 beta (LC3B) I and II by Western blotting (LC3B II/I ratio) tended to increase at 1 day (p = 0,093) and 4 days (p = 0,066) of immobility in young, but not aged skeletal muscle (compared to pre) (Figure 6, C–D).

### PGC-1 co-activators

**Figure 7:**
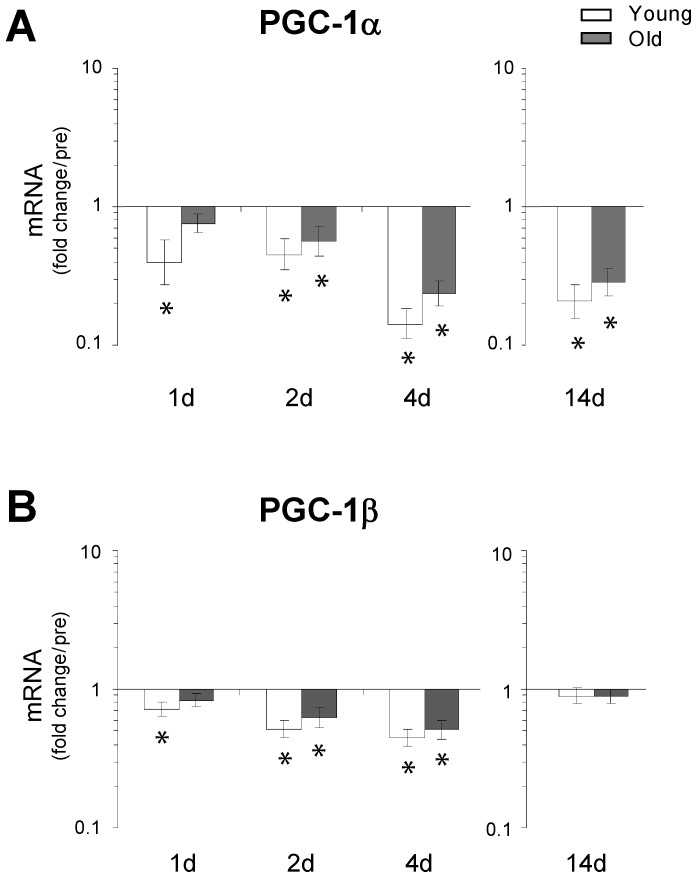
Changes in the transcriptional status of PGC-1α and PGC-1β as a result of immobility-induced disuse-muscle atrophy. The mRNA level of PGC-1α and PGC-1β was determined using qRT-PCR. **A.** The results revealed a marked down-regulation of PGC-1α at 24 h in young muscle and a down-regulation in both young and old muscle at the later time points (2 d, 4 d and 14 d). **B.** The expression levels of PGC-1β mRNA also revealed an age-specific down-regulation after 24 h in young muscle and a down-regulation in both young and old muscle at 2 d and 4 d. In contrast to PGC-1α, the expression levels of PGC-1β mRNA returned to basal levels at 14 days of immobilization, indicating that the two genes may play different roles in the later stages of muscle unloading. * Time effect, p<0.05 compared to pre. Data are geometric means ± back-transformed SEM.

The expression levels of PGC-1α mRNA revealed a marked down-regulation at 1 d in young muscle (Figure 7A) and a down-regulation in both young and old muscle at both intermediate and later time points (2 d, 4 d and 14 d). In line with the present PGC-1α data, expression levels of PGC-1β mRNA revealed a rapid age-specific down-regulation in young muscle following 1 d immobilization, and a more sustained down-regulation in both young and aged muscle at 2 d and 4 d of immobilization (Figure 7B). In contrast, expression levels of PGC-1β mRNA returned to basal levels at 14 d immobilization in both age-groups.

### Apoptosis

**Figure 8:**
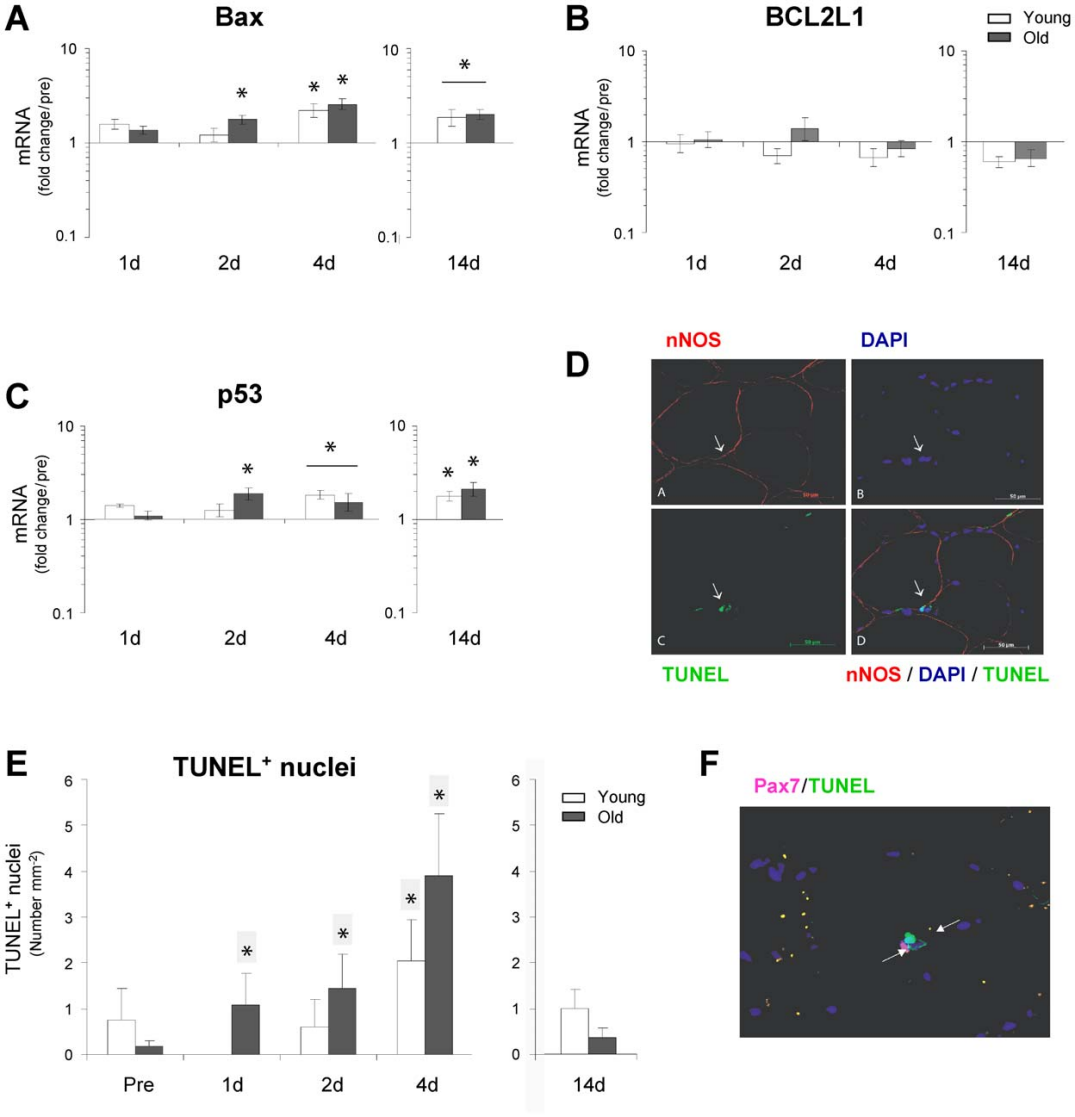
Immobility increases the transcriptional status of Bax, BCL2L1 and p53 and the immunodetection of TUNEL. **A–C**. The mRNA level of Bax, BCL2L1 and p53 was determined using qRT-PCR and significant increases were found in the early phase (0–4 days) as well as later phase (14 days) of disuse-muscle atrophy **D.** Skeletal muscle cryosections were immunostained for nNOS (red), DAPI (blue) and TUNEL (green). **E.** Total numbers of TUNEL-positive nuclei were quantified for both young and old muscle and significant increases of TUNEL-positive nuclei were detected in old muscle in the early phase of immobility (1–2 days) and in both young and old after 4 days of immobility. **F.** Double immunohistochemical staining for TUNEL and the muscle satellite cell marker Pax7 did not reveal any TUNEL-positive muscle satellite cells. Additional green fluorescent expression on the shown image is due to autofluorescence by lipofusin and this is considered non-specific in our analysis. *Time effect, p<0.05 compared to pre. * Time effect, p<0.05 bar indicates young and old combined compared to pre. Data are means ± SEM.

Our data revealed an age-specific up-regulation of Bax and p53 in aged muscle after only 2 days of immobility, with further increasing levels after 4 days in both young and old muscle (Figure 8, A&C). These data were supported by a significant increase in TUNEL-positive nuclei (Figure 8, D–E). However, very few TUNEL-positive myonuclei were observed in both young and old muscle and the expression pattern was not related to the immobilization intervention (data not shown). TUNEL-positive nuclei were mainly localized outside the plasma membrane in the interstitial space between muscle cells, potentially comprised by satellite cells, endothelial cells and leukocytes. Double immunohistochemical staining for TUNEL and the endothelial marker CD-31, as well as the macrophage marker CD-68 and the muscle satellite cell marker Pax7 did not reveal any TUNEL-positive endothelial cells, macrophages (data not shown) or muscle satellite cells (Figure 8F).

### NF-κB signaling and pro-inflammatory cytokines

Apart from a small increase in the expression of NF-κB at 14 d, there was no change in the mRNA expression of NF-κB or TNF-α at any time-point in neither young nor old muscle. Further, there was a small increase in the expression level of IL-6 mRNA in both young and aged muscle after 4 days of immobility, but beside this no major induction of this pro-inflammatory cytokine was observed (Figure S2).

## Discussion

The mechanisms underlying human skeletal muscle atrophy in aged muscle are largely unknown. In the present study, we report transcriptional data from regulatory signaling pathways related to skeletal muscle disuse-atrophy, which has not previously been studied in aging human muscle. The main findings were that irrespectively of age the ubiquitin-proteasome pathway was activated in the very initial phase (2–4 days) of human disuse-muscle atrophy along with a marked reduction in markers of oxidative metabolism. Moreover, an age-specific regulation of Akt and S6 phosphorylation was observed with a decrease in young muscle within the first days (1–4 days) of immobilization. In contrast, aged muscle demonstrated a rise in Akt phosphorylation at 4 days along with a decrease in mRNA expression levels of MuRF-1 and Atrogin-1 after 14 days of leg muscle immobilization. Furthermore, elderly individuals demonstrated less overall muscle loss with disuse than their young counterparts after 14 days (but not 4 days) of muscle disuse. Neither the immediate loss in muscle mass, nor the subsequent age-differentiated signaling responses could be explained by changes in inflammatory mediators or markers of apoptosis.

Certain controversy exists in the literature regarding whether muscle atrophy in human skeletal muscle is regulated primarily via an increase in protein degradation or a decrease in protein synthesis. In animal models, evidence has pointed at protein degradation as the main driving factor, with the ubiquitin-dependent proteolytic system being rapidly activated [3]–[6] in relation to unloading and various disease states [4], [6], [26], although decreases in protein synthesis also have been demonstrated [7], [55], [56]. In contrast, the role of the ubiquitin-proteasome pathway in human in vivo studies has been less consistent [9], [33]–[36], [57]. Our data revealed a significant up-regulation in MuRF-1 and Atrogin-1 within the initial days of immobility (∼2–4 days), with no difference between young and aged muscle. Similar results have recently been observed after 48 h and 72 h of unloading in young human individuals [35], [57], which could suggest a role for the ubiquitin-proteasome pathway in the initiation of human skeletal muscle atrophy. The fact that we observed more modest changes compared to previous animal reports may reflect that more drastic and/or systemic wasting models were used in these animal studies [4]–[6] compared to human immobilization models. Notably, the present data revealed that expression levels of Atrogin-1 and MuRF-1 returned to basal levels after 14 days of immobility in young individuals and was further down-regulated in old individuals, along with a (compared to young) smaller decrease in muscle fiber area. These findings may indicate that the ubiquitin-proteasome pathway mediate a transient rise in protein degradation in human skeletal muscle important for the initial and rapid loss of muscle mass with disuse but may not be important for a more prolonged atrophy response [35]. Notably, a similar time-course of MuRF-1 and Atrogin-1 expression levels has been demonstrated in the rat model after denervation and spinal cord injury [26].

A transient rise in signaling markers of protein degradation does, however, not exclude a simultaneous down-regulation of protein synthesis with immobilization which has been demonstrated to occur in young individuals [9]–[11], [58], [59]. In line with these results, as well as previous data shown by Booth and co-workers in a rat model [7], a decline was observed in phosphorylated Akt and phosphorylated ribosomal protein S6 in the initial phase of immobility (day 1–4) in the present study. In addition to being a central regulator of muscle protein synthesis and muscle hypertrophy the IGF-1/Akt signaling pathway has been proposed to be a potent suppressor of myofibrillar proteolysis and atrophy related ubiquitin ligases, respectively [23], [39], [60], [61]. In speculative terms, the present findings of an age-specific pattern in Akt and ribosomal protein S6 phosphorylation suggests that immobility leads to reduced protein synthesis in young skeletal muscle, in line with previous findings [9]–[11], [58], [59]. In contrast, the - relative to young - higher Akt phosphorylation in elderly in combination with an early up-regulation of MGF and IGF-1Ea expression is potentially contributing to the attenuated atrophy response in aging skeletal muscle observed in the present study. In support of these findings, the expression of molecular markers for anabolic signaling (mTOR and S6K1) and elevated protein synthesis rate either remained unchanged or increased in 24 and 27 months old sarcopenic rats compared to young animals [62].

Although a coordinated regulation of the ubiquitin-proteasome and the autophagy-lysosome pathways has been shown to exist in mice [25], [32], [63], the present study did not demonstrate an increase in expression levels of ATG4, GABARAPL or FoxO3 mRNAs (Figure 6, A–B and Figure 5B). However, we did see a trend towards an increase in LC3B II/I protein ratio selectively in young muscle after 1 d and 4 d of immobility, which suggests that the autophagic process (lipidation) was initiated at least in the young myofibers and thus, crosstalk between the ubiquitin-proteasome and the autophagy-lysosome pathways may also exist in the human model. However, more detailed studies investigating both upstream and downstream regulators of the autophagic and proteolytic processes in humans are needed to elucidate these signaling pathways. Further, the present data revealed that disuse of skeletal muscle resulted in a marked down-regulation of genes involved in oxidative metabolism, consistent with findings from recent human gene array studies [34], [35]. Specifically, our results demonstrated age-specific differences in the modulation of PGC-1α and PGC-1β, with a delayed and smaller response in old muscle compared to that of young individuals. These findings support the hypothesis that the down-regulation of PGC-1α and PGC-1β could be important determinants for the initiation of human skeletal muscle atrophy, as also observed in rodents [27], [41]. Although only minor transcriptional changes of FoxO were noted in the present study, we found a rapid increase in atrogenes downstream of FoxO (Figure 2). However, as protein levels of FoxO were not determined, it is not possible to exclude that high levels of FoxO in the cell and the nucleus will feedback upon the FoxO signaling it self. If so, the present finding of reduced FoxO after 4 days could reflect a feedback phenomenon.

Another topic of debate has been the role of apoptosis in human skeletal muscle atrophy and sarcopenia. There are a significant amount of data indicating an important role for apoptosis in the development of muscle atrophy observed with aging in animal models [14], [42]–[46], whereas human data have been more inconsistent [64]–[66]. Notably, despite an increase in TUNEL-positive nuclei was observed primarily in aging muscle after immobility (Figure 8, D–E), there were limited signs of specific cellular TUNEL-positive myonuclei in young as well as old muscle, in contrast to previous findings in the murine model [67]. The TUNEL-positive nuclei observed in the present study were primarily localized in the interstitial space between muscle cells and the cellular origin and were neither macrophages, endothelial cells nor muscle satellite cells. Thus myofiber as well as muscle satellite cells apoptosis seems not to play a key role for the initiation of human disuse-muscle atrophy in agreement with recent mice studies [68].

In conclusion, the present findings collectively demonstrate that a number of signaling pathways related to both muscle atrophy and muscle hypertrophy are activated in the initial phase of disuse along with a rapid atrophy response in skeletal muscle of both young and old individuals. Importantly, activation of the ubiquitin-proteasome pathway was observed along with a down-regulation of PGC-1α and PGC-1β during the first 1–2 days of disuse, suggesting that proteolysis may play an important role in the initiation of human disuse atrophy in both young and old muscle. These changes were accompanied by rapid decreases in phosphorylated Akt and phosphorylated ribosomal protein S6 selectively in young muscle. In contrary, aged muscle selectively showed an elevated Akt phosphorylation and up-regulation of IGF-1Ea and MGF in combination with a decrease in Atrogin-1 and MuRF-1 mRNA expression levels and a less marked atrophy response in the later phase of disuse.

Although several fundamental mechanistic questions in regards to muscle loss remains to be answered, the present data provide novel insights into the molecular regulation of human skeletal muscle disuse-atrophy and its modulation by aging. Our findings indicate that the initiation and regulation of human skeletal muscle atrophy is age dependent and involves a number of independent signaling pathways. These findings may be important for the identification of biomarkers and future therapeutic intervention paradigms that can be used to counteract human skeletal muscle atrophy in relation to aging and disuse.

## Supporting Information

Figure S1 **Immobility-induced decrease in maximal contractile muscle strength and atrophy of type I and type II muscle fibers.** **A.** Four days of immobility revealed a rapid decrease in maximal contractile muscle in both young and old. The rate of loss in muscle strength seemed to slow down in both groups at 14 d. **B**. The relative decreases in muscle fiber area of type I and type II fibers after 4 d and 14 d of immobility in young and old individuals, revealed a rapid decrease in muscle fiber area of type I as well as type II fibers, respectively. In contrast to young subjects, the rate of muscle loss slowed down in old individuals after 14 d of immobility. * Time effect, p<0.05 compared to pre. # Age effect, p<0.05 difference between young and old within time point. Data are means ± SEM.
(TIF)

Figure S2 **Changes in the transcriptional status of NF-κB, TNF-α and IL-6 as a result of immobility induced disuse muscle atrophy.** The mRNA level of NF-κB, TNF-α and IL-6 was determined using qRT-PCR. **A–B**. A part from a small increase in the expression of NF-κB at 14 d, we did not find any change in the mRNA expression of NF-κB or TNF-α at any time-point in neither young nor old muscle. **C.** A part from a small increase in the expression level of IL-6 mRNA in both young and aged muscle after 4 days of immobility, no major induction of this pro-inflammatory cytokine was observed. * Time effect, p<0.05 compared to pre. * Time effect, p<0.05 bar indicates young and old combined compared to pre. Data are geometric means ± back-transformed SEM.
(TIF)

Figure S3 **Unchanged actin protein levels during skeletal muscle disuse-atrophy.** **A.** Western blotting of whole muscle protein isolates; quantified in **B.** Protein levels of actin were unchanged at the early (2–4 days) phase of immobility in both young and aged skeletal muscle. Data are geometric means ± back-transformed SEM. Due to lack of muscle tissue n = 6 (3 young and 3 old) in these analyses.
(TIF)

Figure S4 **Changes in the transcriptional status of GAPDH as a result of immobility induced disuse muscle atrophy.** mRNA expression levels of GAPDH were determined using qRT-PCR. * Time effect, p<0.05 compared to pre. Data are geometric means ± back-transformed SEM.
(TIF)

Table S1 **Subject characteristics.** There was no difference between subjects from the 14 days and 4 days immobilization study with respect to age, weight and BMI, young and old respectively, however, old subjects weighed more and had a higher body mass index (BMI) than young subjects. # Age effect, p<0.05 old compared to young within time point. Data are means ± SEM.
(TIF)

Table S2 **Primers for the qRT-PCR and TaqMan Low Density array (LDA) assay ID.** **A.** The mRNA expression of FoxO1, FoxO3, FoxO4, PGC-1α, PGC-1β, IL-6, MGF, IGF-1Ea and RPLP0 were analyzed by quantitative real-time RT-PCR. **B.** TaqMan based quantitative real-time RT-PCR of MuRF-1, Atrogin-1, NF-κB, Bax, BCL2L1, p53, TNF-α, ATG4B, GABARAPL1, and RPLP0 mRNA were performed in the ABI Prism 7900HT Sequence System (Applied Biosystems) using ABI TaqMan Low Density Arrays (Applied Biosystems).
(TIF)
