# Supplementary figures and images for: Aging Affects the Transcriptional Regulation of Human Skeletal Muscle Disuse Atrophy
Source: PLoS One. 2012 Dec 19;7(12):e51238. doi: 10.1371/journal.pone.0051238 (PMC3526599; doi:10.1371/journal.pone.0051238)

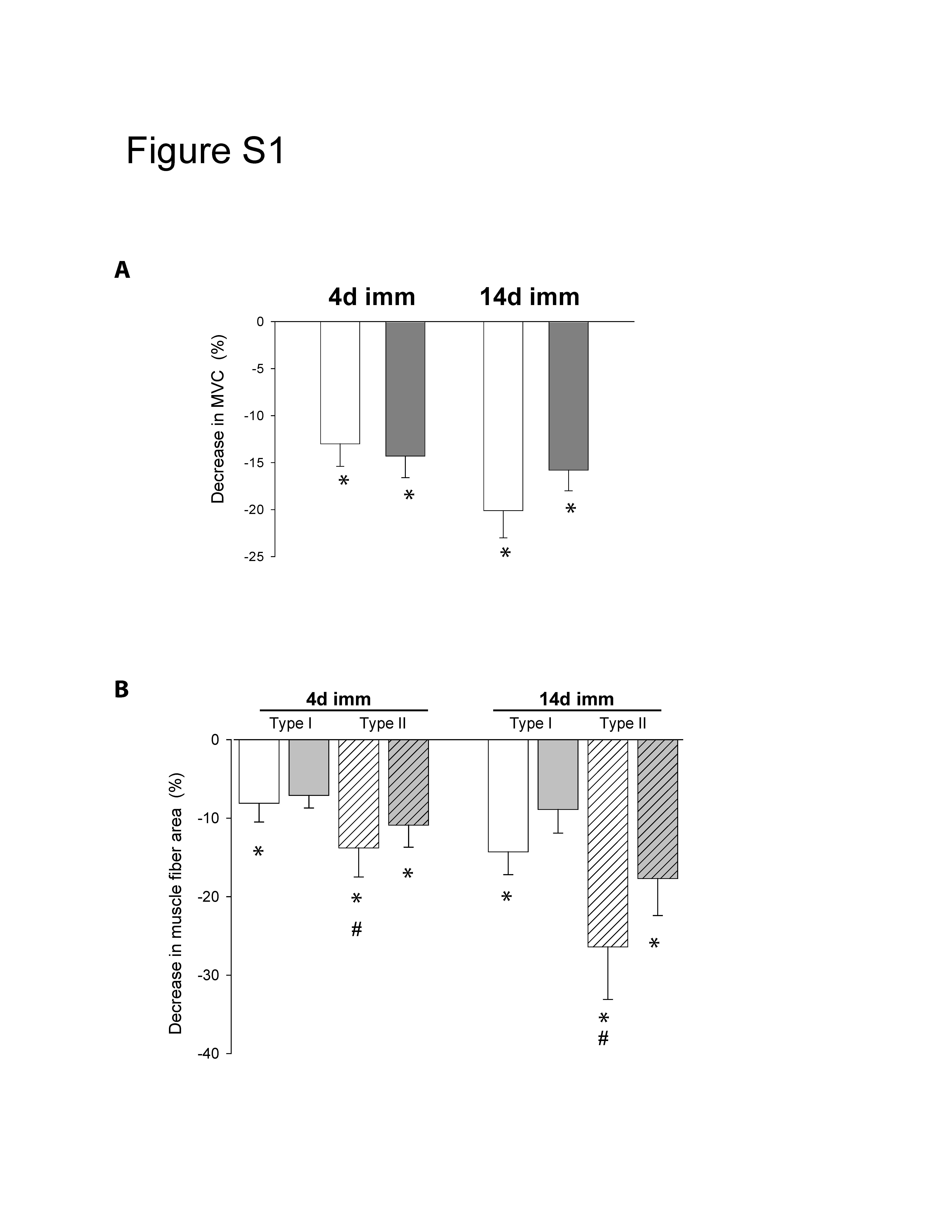

Supplement: Figure S1 — Immobility-induced decrease in maximal contractile muscle strength and atrophy of type I and type II muscle fibers. A. Four days of immobility revealed a rapid decrease in maximal contractile muscle in both young and old. The rate of loss in muscle strength seemed to slow down in both groups at 14 d. B. The relative decreases in muscle fiber area of type I and type II fibers after 4 d and 14 d of immobility in young and old individuals, revealed a rapid decrease in muscle fiber area of type I as well as type II fibers, respectively. In contrast to young subjects, the rate of muscle loss slowed down in old individuals after 14 d of immobility. * Time effect, p<0.05 compared to pre. # Age effect, p<0.05 difference between young and old within time point. Data are means ± SEM. (TIF) [file pone.0051238.s001.tif]

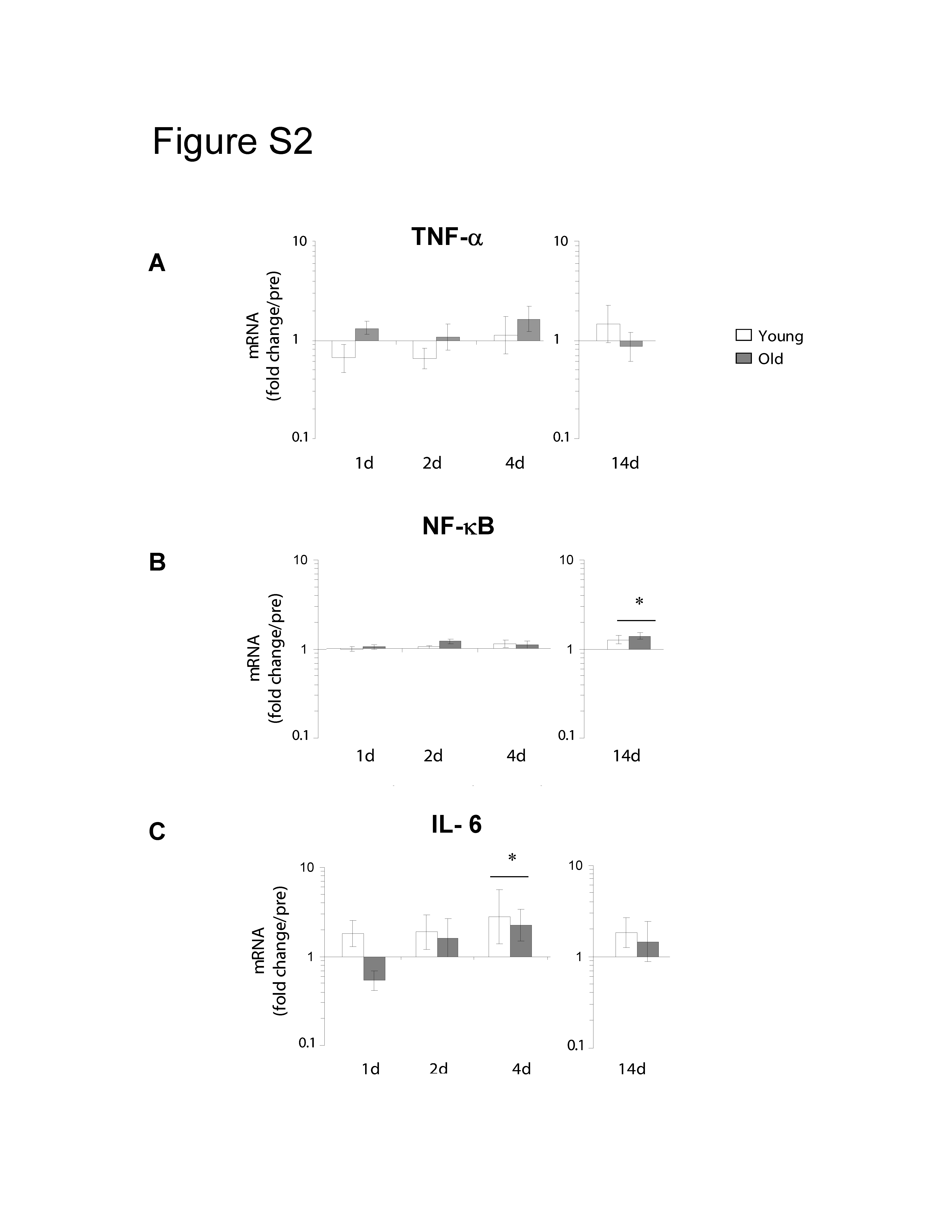

Supplement: Figure S2 — Changes in the transcriptional status of NF-κB, TNF-α and IL-6 as a result of immobility induced disuse muscle atrophy. The mRNA level of NF-κB, TNF-α and IL-6 was determined using qRT-PCR. A–B. A part from a small increase in the expression of NF-κB at 14 d, we did not find any change in the mRNA expression of NF-κB or TNF-α at any time-point in neither young nor old muscle. C. A part from a small increase in the expression level of IL-6 mRNA in both young and aged muscle after 4 days of immobility, no major induction of this pro-inflammatory cytokine was observed. * Time effect, p<0.05 compared to pre. * Time effect, p<0.05 bar indicates young and old combined compared to pre. Data are geometric means ± back-transformed SEM. (TIF) [file pone.0051238.s002.tif]

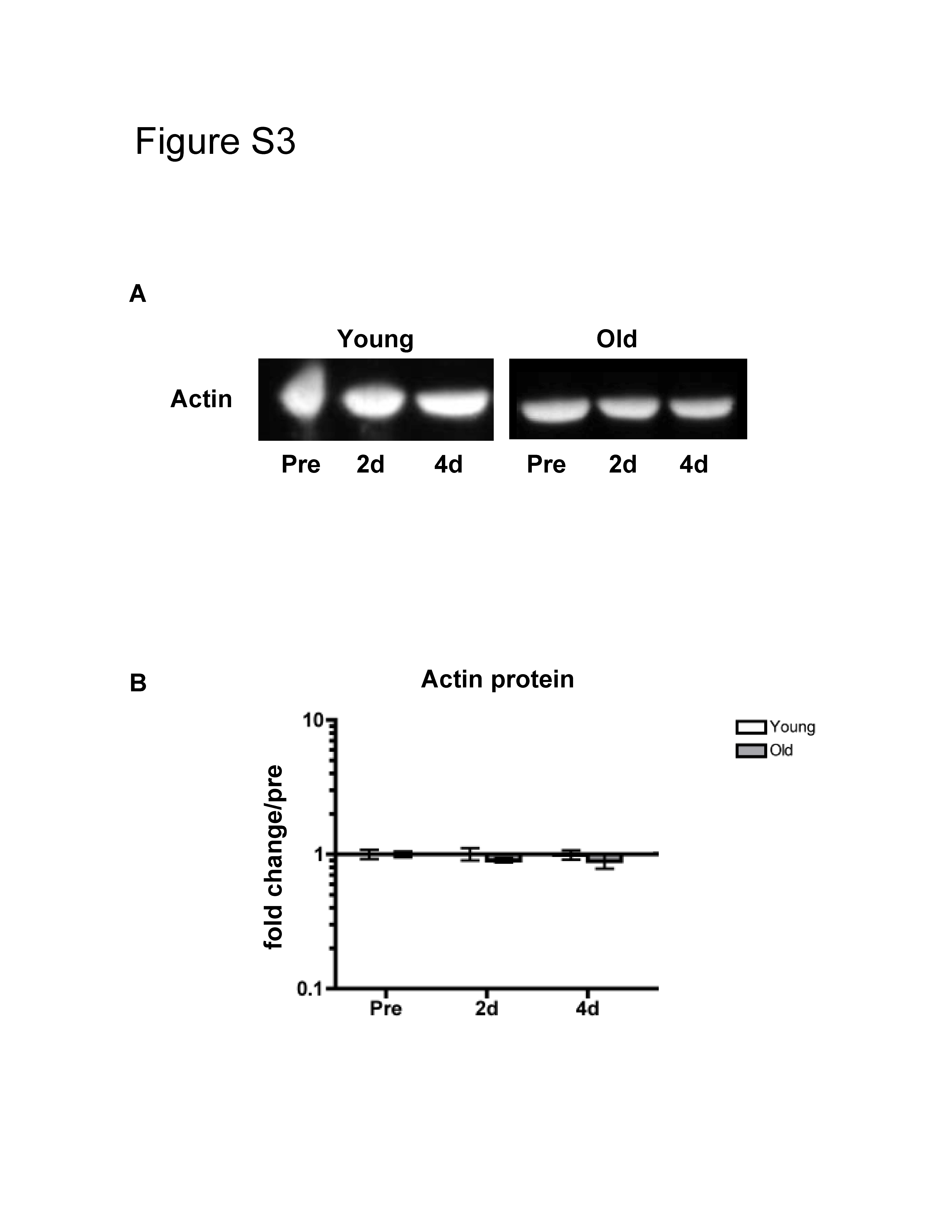

Supplement: Figure S3 — Unchanged actin protein levels during skeletal muscle disuse-atrophy. A. Western blotting of whole muscle protein isolates; quantified in B. Protein levels of actin were unchanged at the early (2–4 days) phase of immobility in both young and aged skeletal muscle. Data are geometric means ± back-transformed SEM. Due to lack of muscle tissue n = 6 (3 young and 3 old) in these analyses. (TIF) [file pone.0051238.s003.tif]

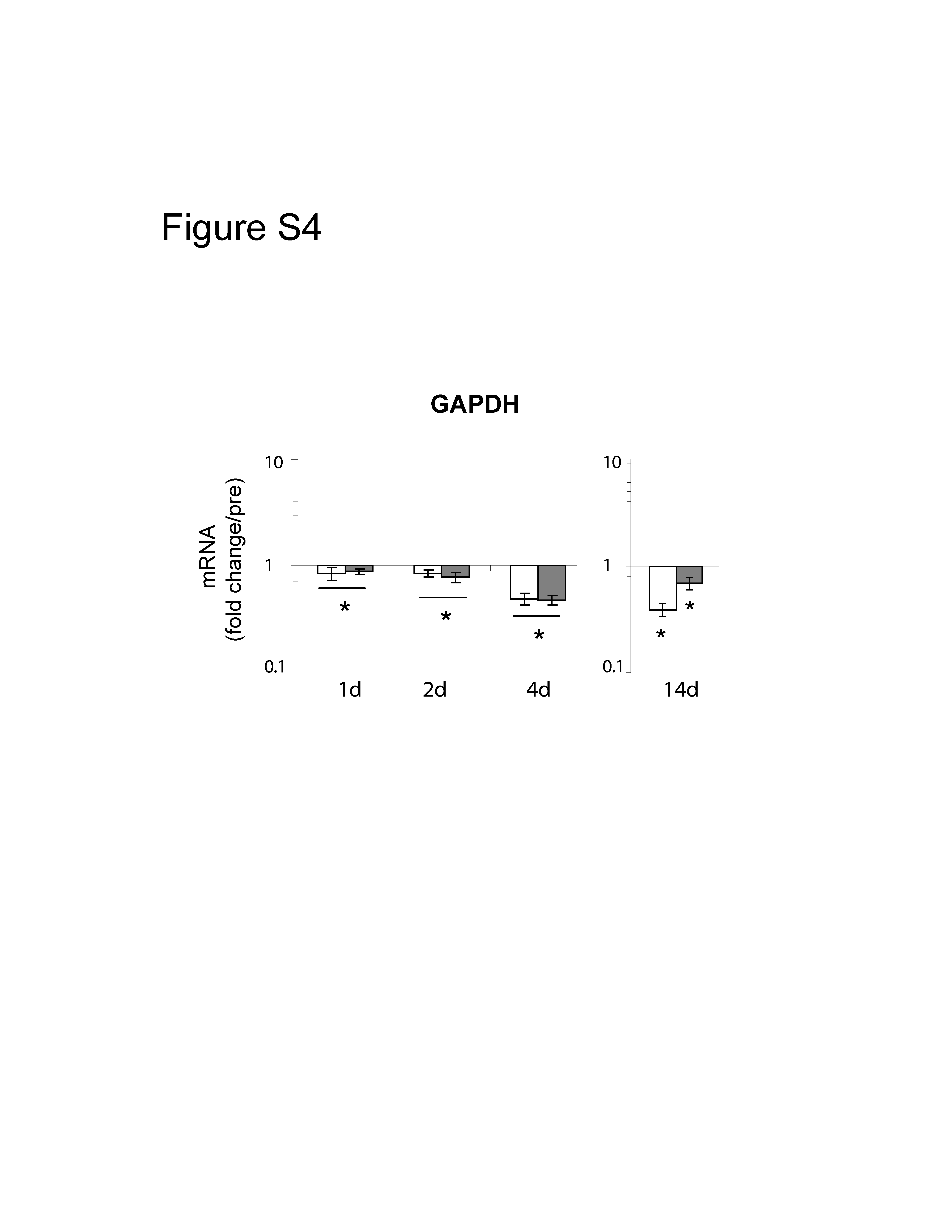

Supplement: Figure S4 — Changes in the transcriptional status of GAPDH as a result of immobility induced disuse muscle atrophy. mRNA expression levels of GAPDH were determined using qRT-PCR. * Time effect, p<0.05 compared to pre. Data are geometric means ± back-transformed SEM. (TIF) [file pone.0051238.s004.tif]

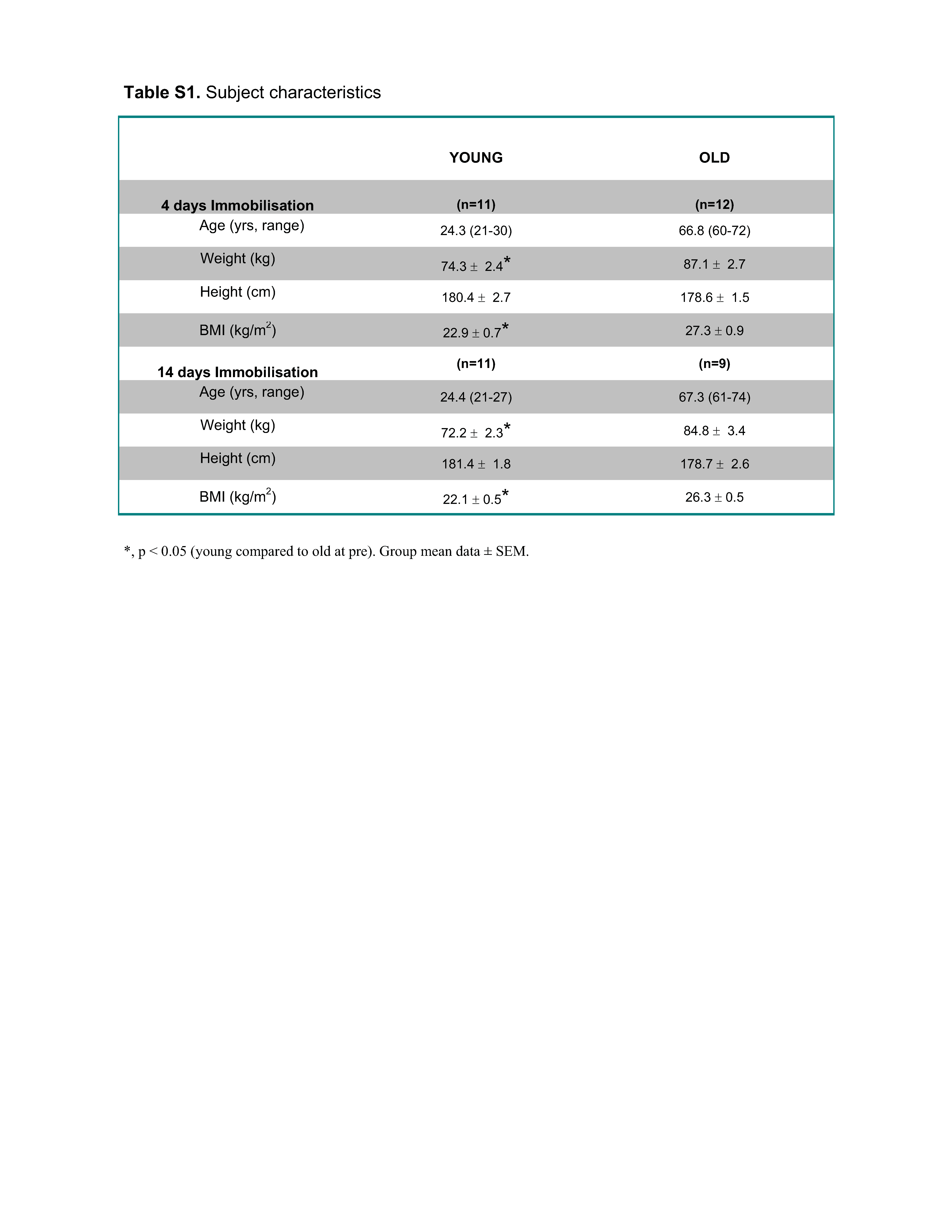

Supplement: Table S1 — Subject characteristics. There was no difference between subjects from the 14 days and 4 days immobilization study with respect to age, weight and BMI, young and old respectively, however, old subjects weighed more and had a higher body mass index (BMI) than young subjects. # Age effect, p<0.05 old compared to young within time point. Data are means ± SEM. (TIF) [file pone.0051238.s005.tif]
